# Associations Between Parity and Parasympathetic Regulation in Response to Infant Crying

**DOI:** 10.1002/dev.70214

**Published:** 2026-10-28

**Authors:** Micol Gemignani, Hanneli Sinisalo, Mikko J. Peltola

**Affiliations:** ^1^ Department of Psychology and Cognitive Science University of Trento Rovereto Italy; ^2^ Social Services and Health Care Tampere University of Applied Sciences Tampere Finland; ^3^ Human Information Processing Laboratory, Faculty of Social Sciences Tampere University Tampere Finland

**Keywords:** arousal, emotion, heart rate, infant crying, parenting, parity, RSA

## Abstract

Research suggests that women's parasympathetic response to infant crying reflects regulation of arousal and is associated with caregiving behaviors. In particular, respiratory sinus arrhythmia (RSA) withdrawal in response to infant cry stimuli has been associated with maternal sensitivity. However, the ways in which multiple reproductive and caregiving experiences (in terms of giving birth and raising a child) can modulate women's RSA in response to infant crying are still unknown. Our aim was to test whether women's RSA in response to infant crying is associated with parity. Participants included 184 women (62 nulliparous women; 86 primiparous women; 36 multiparous women). RSA was measured while women were watching a neutral video, followed by videos of crying infants. Analyses revealed that perception of infant crying was associated with a significant RSA withdrawal, compared to the baseline condition, in both multiparous and nulliparous women. A statistically significant RSA withdrawal was absent among primiparous women, possibly reflecting a more sustained physiological arousal to infant crying. We discuss the potential challenges in physiological regulation, indexed by RSA withdrawal, among primiparous women in response to infant crying. More sustained arousal may support an increased focus on infant signals but also predispose new mothers to perceive greater stress.

## 1 Introduction

Vigilance and reactivity to infant cues facilitate sensitive caregiving behaviors, which contribute to the formation of a secure attachment relationship between a parent and a child (Ainsworth et al. 1978). Infant crying constitutes a potent stimulus that elicits an immediate caregiving response in parents, alerting them to the infant's need for care (Bell and Ainsworth 1972, Bowlby 1969/1982). To respond to infants’ crying in an optimal manner, parents must be able to manage their own emotional responses in the first place; otherwise, crying can elicit nonoptimal responses or even hostile behaviors (Crouch et al. 2008; Leerkes et al. 2023). In recent years, there has been a growing interest in how parents’ responses to infant crying are associated with the quality of caregiving. For instance, prior research has found that mothers who exhibit more optimal emotional and physiological responses to infant crying tend to interact more sensitively with their infants (Leerkes et al. 2016, 2023). Nonetheless, despite its importance for appropriate care, it remains unclear whether and how physiological responses to infant crying change across different stages of parenthood.

Infant crying effectively promotes caregiving behaviors in adults, partly by increasing their physiological arousal (Joosen, Mesman, Bakermans‐Kranenburg, & van IJzendoorn 2013; Joosen, Mesman, Bakermans‐Kranenburg, Pieper, et al. 2013). One commonly investigated measure of autonomic nervous system activity in adults is *respiratory sinus arrhythmia* (RSA), which reflects heart rate variability across respiratory cycles and is based on parasympathetic control of cardiac activity (Shaffer et al. 2014). The functional role of RSA has often been interpreted in light of the polyvagal theory (Porges 2007), according to which engagement in social relationships is supported by an increased vagal influence on heart rate. In particular, a decrease in RSA (referred to in the literature interchangeably as RSA decrease, withdrawal, or vagal suppression) in response to interpersonal situations is thought to reflect an individual's ability to cope with the demands of a social task. On the other hand, a lack of RSA withdrawal in response to interpersonal situations and external stressors may reflect difficulty in emotional regulation (Porges 1995, 2007, 2021). It is also true that excessive RSA withdrawal to stressors has been linked to diverse forms of psychopathology (e.g., anxiety, phobias, panic disorder, depression) (T. P. Beauchaine 2015), suggesting that there may be an optimal range for RSA response, although no explicit criteria for excessive RSA responses have been established. Even though the polyvagal theory (Porges 1995, 2007, 2021) has evoked much criticism, particularly regarding its anatomical basis (Grossman 2023; Grossman and Taylor 2007), dynamic changes in RSA according to different environmental demands are consistently thought to support interpersonal behaviors (Appelhans and Luecken 2006). Relevant to this study, past research has demonstrated that the amount of RSA withdrawal to stressors is associated with better emotion regulation and behavior control in both children and adults (Balzarotti et al. 2017; Graziano and Derefinko 2013).

When compared to a neutral baseline, exposure to infant crying typically produces a decrease in RSA in mothers (Joosen, Mesman, Bakermans‐Kranenburg, Pieper, et al. 2013; Leerkes et al. 2015). Currently, there are no studies on RSA responses to infant crying in fathers. The ability to regulate physiological arousal, in terms of RSA suppression, however, is likely relevant for all caregivers. Reductions in RSA to infant crying have been found to be more pronounced in highly sensitive mothers (Joosen, Mesman, Bakermans‐Kranenburg, Pieper, et al. 2013), less intrusive mothers (Mills‐Koonce et al. 2009), and mothers with secure attachment representations (Ablow et al. 2013; Xu and Groh 2023). Stronger RSA withdrawal in mothers has been related to more adequate soothing behaviors in response to infant distress during the Still‐Face Paradigm (Ham and Tronick 2006; Moore et al. 2009). Conversely, a more sustained arousal triggered by an infant's crying can interfere with the mother's ability to respond sensitively (Joosen, Mesman, Bakermans‐Kranenburg, & van IJzendoorn 2013). Lin et al. (2019) found that poor emotion regulation was linked to blunted RSA reactivity to infant cry in pregnant women (26–40 weeks of gestation). In the same study, a flattened physiological response in mothers was also linked to poorer developmental outcomes for infants. Groh et al. (2019) evidenced that lower maternal RSA withdrawal during a distressing caregiving task predicted greater infant avoidance (indicating infant attachment insecurity) when infants were 12 months old. Speck et al. (2023) used a longitudinal design and found that infants at 7 months exhibited higher levels of avoidance when their mothers displayed lower RSA reactivity to infant crying during pregnancy.

Although maternal ability to regulate physiological responses to infant crying seems to be crucial (Joosen, Mesman, Bakermans‐Kranenburg, Pieper, et al. 2013; Ablow et al. 2013; Speck et al. 2023), it is not yet known whether and how multiple reproductive and caregiving experiences, in terms of giving birth and raising children, modulate women's RSA responses to infant crying. In the present study, these experiences were operationalized through parity, defined as the number of children a woman has had. Indeed, child‐rearing experiences may contribute to short‐ and long‐term neurobiological and hormonal changes supporting more efficient parental care (Bridges 2015). In animal studies, maternally experienced rats, as compared to inexperienced rats, show a greater *fos* expression (marker of neural activity) in the medial preoptic area (MPOA; a region thought to facilitate maternal behavior) when exposed to pups in the days after birth (Fleming and Korsmit 1996). On the other hand, a lower *fos* expression in the medial amygdala (a region thought to inhibit maternal behavior) has been found in maternally experienced rats in the postpartum period (Morgan et al. 1999). This evidence suggests a differential neurobiological profile in rats based on prior reproductive experiences.

In human studies, parity was demonstrated to modulate electrophysiological (EEG) responses to infant cues (Vuoriainen et al. 2022) during pregnancy and early postpartum (Maupin et al. 2016, 2019). Maupin et al. (2019) found that, at 2 months postpartum, primiparous (i.e., first‐time) mothers had a larger P300 amplitude in response to infant emotional faces than multiparous mothers (i.e., those with two or more children), indicating greater attentional engagement to infant emotion cues in first‐time mothers. Considering that smaller ERP amplitudes can reflect more efficient neural processing (Lowell et al. 2023), multiparous mothers might be more efficient in responding to infant emotional cues than primiparous mothers. This effect of parity was found to persist at 7 months postpartum (Bunderson et al. 2020). Pérez‐Hernández et al. (2021) similarly suggested that multiple caregiving experiences lead to greater individual efficiency and effectiveness in responding to infant emotional stimuli. However, Rutherford et al. (2019) found that a heightened allocation of attention in first‐time mothers may not be specific to infant faces, but directed to all visual stimuli, suggesting a general state of alertness in primiparous mothers. In addition, none of the aforementioned studies included nonparents as a control group. To account for different levels of caregiving experience in identifying pain in infant cry, Corvin et al. (2022) conducted psychoacoustic experiments including (1) nonparents with no experience with infants, (2) nonparents with moderate nonprofessional experience (caring for younger siblings), (3) nonparents with extensive professional experience, (4) parents with infants less than 2 years old, and (5) parents with children of at least 5 years. The findings suggest that increased caregiving experience enhances individuals’ ability to use acoustic information to identify pain in infant cries. In other words, adults with more experience with infants seem to have a better capacity to decode the information conveyed by infant cries (Corvin et al. 2022).

### 1.1 Current Study

The aim of the present study is to investigate women's RSA response to infant crying as a function of parity, by examining primiparous mothers, multiparous mothers, and a control group of nulliparous (nonparent) women. The participants’ heart rate was recorded while they were watching a neutral baseline video, followed by video clips of crying infants. In line with previous findings (Joosen, Mesman, Bakermans‐Kranenburg, Pieper, et al. 2013; Leerkes et al. 2015), we expected that exposure to the cry stimuli would produce a decrease in RSA from the neutral baseline. Regarding the role of parity, we expected that multiparous women would demonstrate a greater RSA decrease to cry stimuli compared to primiparous women, given previous findings indicating a more efficient processing of infant emotion cues in more experienced mothers (Maupin et al. 2019; Bunderson et al. 2020; Pérez‐Hernández et al. 2021). Although there is a substantial lack of previous research on nonmothers, they could be expected to also show an RSA decrease to infant cry, given the stressful nature of the cry stimuli. We did not, however, formulate explicit a priori hypotheses regarding whether the RSA responses of nulliparous women would significantly differ from the two groups of mothers. Given that autonomic reactivity may also vary as a function of individual characteristics, we preliminarily examined whether the groups differed in factors that could contribute to RSA responses to infant crying. Together with age and other relevant background characteristics, particular attention was given to anxiety, due to its established association with RSA reactivity (T. P. Beauchaine 2015) and its relevance to maternal responses to infant cues (Guo et al. 2018). In addition, we calculated heart rate levels (beats per minute; BPM) across the baseline and cry conditions. As an index of sympathetic cardiac arousal, we used heart rate measurement to complement the RSA analysis by testing whether the presentation of cry stimuli would increase participants’ arousal levels (i.e., by increasing heart rate; Frodi et al. 1981) and explore whether heart rate reactivity (i.e., the expected increase) is influenced by parity.

## 2 Methods

### 2.1 Participants

The sample consisted of 184 Finnish women who participated in the TransParent project, which investigated changes in processing infant cues during the transition to parenthood. They were either nulliparous (*n* = 62, *M*_age_ = 26.2 years, SD_age_ = 3.4), primiparous (*n* = 86, *M*_age_ = 31.0 years, SD_age_ = 3.9), or multiparous (*n* = 36, *M*_age_ = 33.8 years, SD_age_ = 5.5) women. Two additional participants were recorded but excluded because of excessive artifacts in the heart rate data (*n* = 1) or for being pregnant at the time of the laboratory visit (*n* = 1). All mothers were recruited by invitation letters based on their contact information obtained from the Finnish Digital and Population Data Services Agency; nulliparous women were recruited via university email lists. All participants were in a couple relationship for longer than 6 months, lived together with their partner, and were fluent in Finnish. Mothers had at least one child of around 6 months of age (*M*_age_ = 7.1 months, SD_age_ = 1.1), and nonmothers did not have children of their own or of their partner.

The data reported here were combined from two substudies conducted as part of the TransParent project. The first study (data collected between May 2018 and May 2019) included all 62 nulliparous women and 53 of the 86 primiparous mothers, while the second study (data collected between December 2022 and May 2023) included the remaining 33 primiparous mothers and all 36 multiparous mothers. For the first study, in which the main goal was to compare nulliparous and primiparous women with various measures (Sinisalo et al. 2022, 2025), we aimed to recruit a sample of 120 participants (60 in both the nulliparous and the primiparous groups) to obtain sufficient power for a 2 × 2 mixed ANOVA design. In the second study, designed to compare primiparous and multiparous mothers, the targeted sample size of 70 participants (35 participants in both the primiparous and the multiparous groups) was slightly smaller due to time constraints imposed on the project by the Covid‐19 pandemic. The primiparous group therefore consisted of participants from both substudies (i.e., before and after the pandemic). It is important to note, however, that primiparous participants were recruited using the same criteria in both substudies. We checked for any potential differences between these two batches of participants in measures that were included in both substudies (i.e., RSA and anxiety questionnaire scores). No differences between these two batches of participants in these measures were found, supporting their pooling for the main analyses. The research protocols for both substudies were approved by the Ethics Committee of the Tampere Region (protocol numbers 16/2018 and 97/2022). The study was conducted in accordance with the Declaration of Helsinki. All participants provided informed written consent before participating.

### 2.2 Procedure

Whenever possible, the laboratory visits were scheduled in the luteal phase of the menstrual cycle as an attempt to control for variation in hormonal levels across the menstrual cycle. Participants were asked not to eat or drink for a full hour before the visit. Similarly, breastfeeding mothers were asked to breastfeed their baby 1 h before the visit. In both substudies, the RSA recording protocol was identical except for slight differences in recording equipment (see below), and the RSA recording was similarly scheduled midway through a series of tasks and assessments. Within a few days after the laboratory visit, participants received a link via email to an online set of questionnaires.

To measure RSA responses to infant cry stimuli, participants were provided with headphones and asked to sit still in front of a 19‐inch screen and watch a series of videos for approximately 6 min. First, a video of a woman quietly assembling a tower of blocks (Peltola et al. 2017) was presented for 145 s to obtain a resting RSA baseline measurement. Next, after a break of 15 s, three consecutive videos of crying infants (total duration of 145 s) were presented to measure RSA responses to infant cry. The cry videos (Wass et al. 2018) displayed young (<6 months) infants either in a supine or sitting position in a natural context (on a blanket with no other people or salient visual features present), crying with moderate to high intensity throughout the video. After the cry videos, a 90‐s positive video of an infant waking up and smiling was presented to alleviate potential negative affect triggered by the cry videos.

### 2.3 Heart Rate Measurement and Analysis

During the presentation of the videos, electrocardiograph (ECG) data were continuously collected using two Ag/AgCl electrodes on the left and right sides of the upper chest area, with a ground electrode placed on the forehead (first substudy) or in the back of the neck (second substudy). ECG was recorded with BrainVision Recorder software through a Brain Products QuickAmp (first substudy) or an actiCHamp Plus amplifier (second substudy) at a sampling rate of 1000 Hz.

Offline, the ECG data were processed with ECGtool, an in‐house, MATLAB‐based software to identify QRS complexes in the ECG signal and to measure the time between two successive R‐peaks (i.e., interbeat intervals, IBIs). The R‐peaks were first detected automatically and then manually corrected, when necessary, for falsely detected or omitted R‐peaks. The IBIs within the baseline and cry video segments were converted to BPM to compare average heart rate levels between the segments. To calculate RSA, the IBI series were first converted to a time series sampled at 10 Hz by linear interpolation and filtered using a 241‐point optimal finite impulse response bandpass filter with a 0.12–0.40 Hz frequency band. RSA was then calculated as the natural logarithm of the variance of the filtered signal (Allen et al. 2007) separately for the neutral baseline video and the cry videos.

### 2.4 Questionnaire Measures

For all participants, the self‐report measures consisted of items covering background information such as participant age and education years. For mothers, information was also collected about child age, number of children, breastfeeding status (yes/no), and the time (min) passed since their last breastfeeding. For nulliparous women, additional questions concerned regular nonparental caregiving experiences of children under 2 years, such as caring for others’ children or siblings (yes/no). For all participants, the State‐Trait Anxiety Inventory (STAI; Spielberger et al. 1983; Bieling et al. 1998) was also administered to measure general anxiety symptoms. In the first substudy, a six‐item STAI was used (Spielberger et al. 1983), while in the second substudy, the standard 20‐item version was adopted. To make sure that the two scales were comparable, the sum scores of the six‐item scale (Bieling et al. 1998) were multiplied by 3.33 (i.e., 20/6).

### 2.5 Statistical Analyses

The statistical analyses were performed using RStudio 2022.12.0. Descriptive statistics were run to examine mean scores, frequencies, percentages, and distributions of the variables. Missing data were not replaced. Normality assumptions were checked using the Shapiro–Wilk test. Bartlett's test was used to check equality of variances. Pre‐existing group differences in terms of age, education years, time passed since breastfeeding, and anxiety symptoms (STAI) were tested through one‐way ANOVAs. Pearson's Chi‐squared test was used to test differences between primiparous and multiparous women in the categorical variable (breastfeeding: yes/no). A repeated‐measures ANOVA predicting RSA among the three groups (group: between‐subject factor) and within conditions (condition: within‐subject factor; baseline vs. cry condition) was performed, with age as a covariate due to the groups differing in age (see Section 3.1). Main effects were interpreted before the interactions. Follow‐up analyses were conducted to examine changes in RSA across conditions within each group, as well as group differences separately within the baseline and cry conditions. Post hoc analyses were corrected using the Tukey method. Effect sizes of ANOVA models were reported using the partial eta squared (*η*_p_^2^). Exploratory analyses were conducted to test whether differences in the time passed since breastfeeding in mothers (see Section 3.1) and nonparental caregiving experience among nulliparous women were associated with the RSA responses. To complement the RSA analyses, a separate repeated‐measures ANOVA predicting heart rate (BPM) was conducted using the same between‐subject factor (group), within‐subject factor (condition: baseline vs. cry condition), and age as a covariate. An anonymized version of the data is available at https://osf.io/sny8j/.

## 3 Results

### 3.1 Preliminary Analysis

Characteristics of the study participants, heart rate, and RSA data are reported in Table 1. Preliminary differences among the groups emerged in terms of age (*F*(2, 181) = 44.3, *p* < 0.001; *η*_p_^2^ = 0.3, 95% CI [0.24, 1.00]); multiparous women were older than primiparous (diff = 2.7, 95% CI [1.1, 4.3], *t* = 3.3, df = 181, *p* = 0.003) and nulliparous (diff = 7.6, 95% CI [5.9, 9.2], *t* = 8.8, df = 181, *p* < 0.001) women. Moreover, primiparous women were older than nulliparous women (diff = 4.8, 95% CI [3.5, 6.2], *t* = 7.1, df = 181, *p* < 0.001). Among the women who declared to breastfeed, the time since last breastfeeding differed statistically significantly (*F*(1, 99) = 4.1, *p* < 0.05; *η*_p_^2^ = 0.04, 95% CI [0.01, 1.00]); primiparous women exhibited a shorter time since their last breastfeeding compared to multiparous women (diff = −186, 95% CI [−313.8, −58.9], *t* = −2.0, df = 99, *p* < 0.05). No statistically significant differences were found among the three groups in the years of education (*p* = 0.08) or (STAI) anxiety symptoms (*p* = 0.2). Similarly, no statistically significant differences emerged between primiparous and multiparous women in child age (*p* = 0.6) or breastfeeding status (yes/no) (*p* = 0.4).

**TABLE 1:** Descriptive statistics of participants by groups; *n* = number; *M* = mean; *P* = percentage; SD = standard deviation.

	Nulliparous (*n* = 62; 33.7%)	Primiparous (*n* = 86; 46.7%)	Multiparous (*n* = 36; 19.6%)
Variable	*M* (*SD*) or *P*	*M* (*SD*) or *P*	*M* (*SD*) or *P*
Parent age (years)	26.2 (3.4)	31.0 (3.9)	33.8 (5.5)
Child age (months)		7.1 (1.2)	7.0 (0.6)
Number of children			2.2 (0.5)
Education years	16.2 (2.0)	17.3 (2.5)	16.7 (3.6)
Breastfeeding (yes)		80% (*M* minutes from breastfeeding: 80.0)	89% (*M* minutes from breastfeeding: 283.8)
Regular nonparental caregiving experience (yes)	36%		
Anxiety symptoms	37.1 (11.5)	34.1 (9.7)	35.3 (7.8)
Heart rate (BPM)			
BPM baseline	71.5 (10.6)	71.5 (8.5)	74.2 (8.3)
BPM cry	72.8 (10.9)	72.7 (9.5)	75.7 (8.2)
Respiratory sinus arrhythmia (RSA)			
RSA baseline	6.5 (0.9)	6.1 (0.9)	6.1 (0.8)
RSA cry	6.2 (1.0)	6.0 (0.9)	5.8 (0.9)

### 3.2 Main Analysis

The Shapiro–Wilk test confirmed the normality assumption of RSA data (*p* = 0.4). The variance of RSA in each group tested equal (*p* = 0.5). A repeated‐measures ANOVA predicting RSA within conditions and between groups evidenced a main effect of condition (*F*(1, 181) = 18.7, *p* < 0.001; *η*_p_^2^ = 0.09, 95% CI [0.04, 1.00]); as expected, exposure to cry videos was associated with a decreased RSA (diff = −0.19, 95% CI [−0.26, −0.11], *t* = −4.7, df = 181, *p* = 0.003) compared to the baseline condition. A main effect of group in RSA values was also found (*F*(2, 180) = 3.5, *p* = 0.03; *η*_p_^2^ = 0.04, 95% CI [0.00, 1.00]); however, post hoc analyses did not show any statistically significant contrasts (all *p*s > 0.05), indicating no clear differences in the overall RSA values between groups. Women's age was a statistically significant covariate (*F*(1, 180) = 15.0, *p* < 0.001; *η*_p_^2^ = 0.08, 95% CI [0.03, 1.00]); higher RSA levels were observed in younger women compared to older women. An interaction effect between condition and group was also evidenced (*F*(2, 181) = 3.7, *p* = 0.03; *η*_p_^2^ = 0.04, 95% CI [0.00, 1.00]); the cry condition was associated with a significantly lower RSA than the baseline condition in multiparous (diff = −0.25, 95% CI [−0.41, −0.08], *t* = −3.0, df = 181, *p* = 0.04) and nulliparous (diff = −0.26, 95% CI [−0.38, −0.13], *t* = −4.0, df = 181, *p* = 0.001) women. However, no statistically significant change in RSA from the baseline to the cry condition was detected in primiparous women (*p* = 0.9). A graphical representation of these group differences is displayed in Figure 1. As an additional follow‐up analysis, we explored group differences within the baseline and cry conditions. Within the baseline condition, a main effect of the group was found (*F*(2, 180) = 5.0, *p* = 0.008; *η*_p_^2^ = 0.05, 95% CI [0.01, 1.00]); however, post hoc analyses did not show any statistically significant differences in baseline RSA values across groups (all *p*s > 0.05). Within the cry condition, a significant effect of the group was not found (*p* = 0.1). Among the women (primiparous and multiparous mothers) who declared to breastfeed, we repeated the RSA model including time since the last breastfeeding. The main effect of condition (*F*(1, 99) = 7.1, *p* < 0.01; *η*_p_^2^ = 0.07, 95% CI [0.01, 1.00]) and the interaction effect between group and condition (*F*(1, 99) = 6.5, *p* = 0.01; *η*_p_^2^ = 0.06, 95% CI [0.01, 1.00]) remained statistically significant, indicating that the observed pattern of RSA responses among groups was not accounted for by time since the last breastfeeding. The cry condition was associated with a significantly lower RSA than the baseline condition in multiparous women (diff = −0.29, 95% CI [−0.45, −0.13], *t* = −3.6, df = 99, *p* = 0.006). Again, a similar decrease was not detected in primiparous women (*p* = 0.9). Within the group of nulliparous women, we examined whether regular nonparental caregiving experience (yes/no) was associated with RSA differences across conditions, but no influence of caregiving experience within this group was detected (*p* = 0.2). In addition, we ran a repeated‐measures ANOVA predicting BPM within conditions and between groups. A statistically significant main effect of condition emerged (*F*(1, 179) = 38.8, *p* < 0.001; *η*_p_^2^ = 0.1, 95% CI [0.10, 1.00]), indicating an increase in heart rate from baseline to the cry condition (diff = 1.34, 95% CI [0.90, 1.78], *t* = 6.0, df = 179, *p* < 0.001) across all participants, while no significant main effect of group (*p* = 0.3), age (*p* = 0.8), or interaction between group and condition (*p* = 0.9) was found (see Figure 2 for illustration).

**FIGURE 1:**
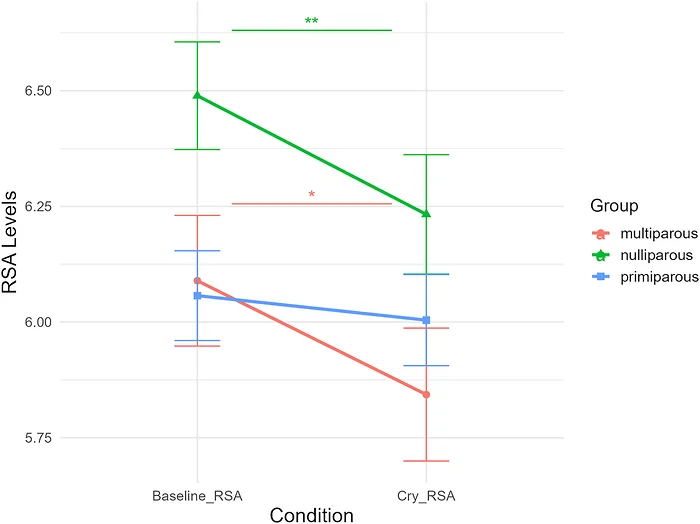
Mean RSA levels during the baseline and cry conditions by group (multiparous, nulliparous, primiparous women). Error bars = ±1 SE. Asterisks denote statistically significant within‐group differences between conditions (*p < 0.05; **p < 0.01).

**FIGURE 2:**
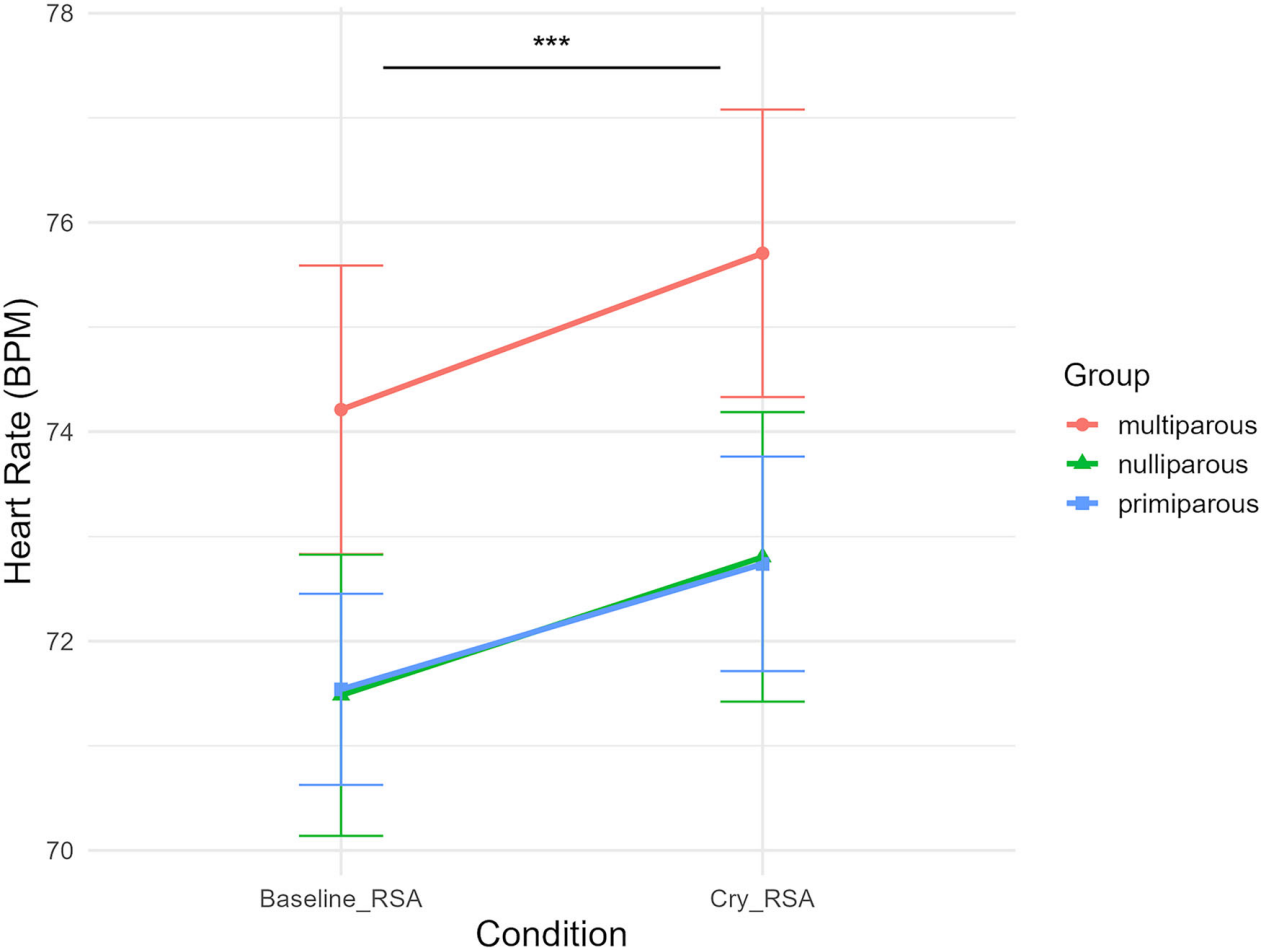
Mean heart rate (BPM) during the baseline and cry conditions by group (multiparous, nulliparous, primiparous women). Error bars = ±1 SE. Asterisks denote the statistically significant increase in heart rate from baseline to cry conditions across all groups (***p < 0.001).

## 4 Discussion

The aim of this study was to examine whether and how women's physiological regulation in response to infant crying, as indexed by RSA withdrawal, was associated with parity. Our results showed that both nulliparous and multiparous women had a statistically significant RSA withdrawal from baseline to infant cry stimuli. The same effect was not detected in primiparous women. Adopting a psychobiological perspective, this study adds meaningful knowledge on the pathways by which women's response to infant distress can be influenced by different stages of parenthood, pointing to a potentially increased vulnerability to stress in first‐time mothers.

Overall, RSA levels exhibited the expected pattern of change from baseline to infant crying. The validity of the experimental task was further confirmed by increased heart rate (BPM) levels from baseline to cry condition, indicating that the cry videos increased autonomic arousal across the sample. The observed pattern of parasympathetic withdrawal supports the initiation of a behavioral response to infant crying and regulation of overarousal (T. Beauchaine 2001; Berntson et al. 1991). In line with previous studies showing differences in the neural responses to infant faces (Maupin et al. 2016, 2019) and in the evaluation of infant cry sounds (Corvin et al. 2022) as a function of parity, we found that parity influenced women's physiological response to infant crying, independently of covariates. As indicated by the preliminary analyses, no pre‐existing differences in general anxiety among the groups could account for the observed RSA differences. While nulliparous and multiparous women displayed a significant RSA withdrawal to infant crying, a similar RSA withdrawal was absent in primiparous women.

Reduced RSA and, on the other hand, excessive RSA reactivity to emotional challenges have been observed among individuals with poorer emotion regulation abilities (T. P. Beauchaine 2015). Therefore, the pattern evidenced here might be associated with a less adaptive physiological response to infant distress in primiparous mothers, indicating that they can respond to infant distress with a more sustained (i.e., not downregulated) arousal. While such heightened arousal may be adaptive in the sense that it increases the perceived urgency of infant crying in new mothers (Bell and Ainsworth 1972), it may also predispose them to misinterpret infant crying signals, experience crying as more aversive, and focus on their own needs to cope (Leerkes et al. 2015). In line with this possibility, a growing number of studies have demonstrated that a lack of RSA withdrawal in response to infant distress can be associated with suboptimal caregiving responses (Joosen, Mesman, Bakermans‐Kranenburg, Pieper, et al. 2013; Leerkes et al. 2015; Ablow et al. 2013; Moore et al. 2009; Gao et al. 2023). Taken as a whole, our findings suggest that adaptive regulation in response to infant crying can be more challenging for first‐time mothers than for experienced mothers. A poorer ability to regulate in this group may reflect greater maternal preoccupation with infants (Bunderson et al. 2020; Winnicott 1956), which supports focusing attention on infant signals, but also raises the risk of suboptimal caregiving behaviors in new parents (Leckman et al. 1999; Kim et al. 2013). It should be noted, however, that the heart rate (BPM) data did not clearly indicate greater arousal levels in primiparous mothers, as we only observed an effect of increased heart rate in the cry condition across the whole sample. That is, the multiparous and nulliparous groups appeared to display a coordinated response pattern of increased sympathetic arousal coupled with parasympathetic withdrawal supporting behavioral responses to the stressor (Berntson et al. 1991), while primiparous mothers only displayed sympathetic arousal. Future studies investigating parity would benefit from incorporating other and potentially more sensitive measures of sympathetic arousal, such as skin conductance level (SCL), as it has been found that SCL and RSA responses to infant crying have interactive effects on maternal sensitivity (Leerkes et al. 2016, 2023). It is also important to acknowledge that the wide confidence interval of the interaction effect between parity and stimulus condition indicates substantial uncertainty in the estimate; this further highlights the need for replication to clarify the robustness of this finding.

Another interpretation of the absence of RSA suppression in primiparous mothers is that the response pattern could instead indicate a more regulated response to infant crying in this group, suggesting that they remained relatively calm when exposed to sustained infant distress. This interpretation is countered, however, by the finding of similar sympathetic arousal to infant cry in primiparous mothers as compared to the other groups. Moreover, as previous research has found primiparous mothers to show greater preoccupation related to caregiving (Kim et al. 2013), more pronounced attention to infant emotion cues (Bunderson et al. 2020), and greater worry and cortisol reactivity in response to observing one's own child in a novel context (Kalomiris and Kiel 2016), first‐time mothers are likely to be more reactive to infant distress signals than multiparous mothers.

Notably, we did not find any statistically significant differences in RSA response between nulliparous and multiparous women. Even though infant cue processing undergoes profound changes over the transition to motherhood (Leuner et al. 2010; Kim et al. 2016; Hoekzema et al. 2017), it could be that, after repeated childbirths, reactivity to infant cues becomes less pronounced in multiparous women, resembling that of women without children. A recent study (Veistola et al. 2025) investigating empathic reactivity in men at different phases of fatherhood supported this interpretation. The study found that affective empathy to children's distress was relatively lower in experienced fathers than in expecting men and in first‐time fathers. Moreover, the question of which aspects of caregiving are actually specific to parents, and which rely on general cognitive abilities, is still under debate (Gustafsson et al. 2013; Bouchet et al. 2020; Gemignani, Giannotti, and de Falco 2024; Parsons et al. 2017). For instance, Bouchet et al. (2020) demonstrated that cry recognition abilities in adults may depend on the degree of experience and exposure to infants, rather than on parental status. Moreover, caregiving is also not restricted to biological parents in the human species; the evolutionary advantages of alloparenting are numerous, including increased human infant survival (Glasper et al. 2019). Therefore, it can be assumed that an adaptive response to infant crying, which supports cooperative breeding, relies on perceptual and cognitive abilities shared by all adults, rather than being exclusive to parents (Bouchet et al. 2020). Furthermore, in the follow‐up analyses, cardiac vagal tone, as indexed by baseline RSA, seemed to differ among the groups. However, none of the post hoc comparisons between the groups were statistically significant. Since no prior studies have examined mixed samples of mothers and nonmothers, we cannot determine whether parental status is associated with differences in baseline RSA levels.

### 4.1 Limitations and Future Directions

The present study should be considered in the light of important limitations. First, our study was cross‐sectional, so we cannot ascertain whether changes in parental status have an influence on RSA reactivity on a within‐person level. Future studies should strive to replicate and extend our findings with longitudinal designs, which would allow more precise estimation of the individual‐level changes in physiological reactivity to infant crying. The relatively small number of multiparous women in the sample also warrants caution in interpreting differences found between the groups. Sample representativeness is important for future research to assess the generalizability of the findings, as we predominantly recruited a fairly nondiverse sample of women with high or medium‐high levels of education. Although the current results were independent of covariates and not influenced by prior differences in anxiety among the groups, there could be various additional factors that may impact autonomic reactivity in the parenting context, such as depressive symptoms, relationship functioning and social support (Carollo et al. 2023), and, in the case of nulliparous adults, future parenting motivation (Sinisalo et al. 2022). Further research on fathers (Carollo et al. 2023) and nonpregnant parents should also be conducted, which would be informative regarding the roles of pregnancy‐induced biological changes and direct caregiving experience in how responsivity to infant distress may change across major life transitions. While there are no studies on paternal RSA responses to infant distress, prior studies have indicated greater hormonal reactivity in new fathers compared to childless men in response to infant cry (Fleming et al. 2002) and greater empathic responses to children's distress signals in first‐time fathers compared to more experienced fathers (Veistola et al. 2025), suggesting that new fathers could be expected to display a similar pattern of autonomic reactivity to infant cry as primiparous women. In this study, differences related to parity were observed between primiparous and multiparous mothers. However, more subtle differences may occur across separate reproductive experiences (e.g., between multiparous women with two vs. three children). Moreover, parity was defined here as each successful generation of offspring, but it remains unclear whether prior unsuccessful pregnancies may also contribute to individual differences in the absence of postpartum caregiving experiences. Although we accounted for regular nonparental caregiving experiences in nonmothers, caregiving experience in mothers was approximated by parity. Future studies should therefore consider other dimensions of caregiving experience, such as involvement in childcare (Gemignani, Giannotti, Rigo, et al. 2024; Gemignani et al. 2022; Giannotti et al. 2025; Rigo et al. 2025), also among mothers. We also did not assess the quality of maternal caregiving in the current study. Future research should thus investigate the ecological validity of our findings by relating physiological reactivity to infant cues in primiparous and multiparous women to the quality of care provided to infants (Leerkes et al. 2016). Such studies may further our understanding of the pathways contributing to sensitive caregiving behaviors across different stages of parenthood, as well as the challenges that mothers may face at each stage. Finally, a potential limitation also concerns the use of videotaped infant crying as the stimulus, as such videos may contain extraneous information that could interfere with the cry signal. In the present study, the videos contained very little external salient visual information that might interfere with the processing of the cry signal; moreover, similar naturalistic videos have been used in other studies to trigger RSA responses (Leerkes et al. 2015; Ablow et al. 2013; Lin et al. 2019; Speck et al. 2023), adding to the comparability of the findings.

## 5 Conclusions

Our results provide initial evidence that parity is associated with women's parasympathetic response to infant crying. If our results are replicated in future research and with additional measures such as SCL, they may suggest a pattern of more sustained arousal to infant crying in first‐time mothers, potentially reflecting greater susceptibility to caregiving‐related stress in less experienced mothers.

## Conflicts of Interest

The authors declare no conflicts of interest.

## Data Availability

An anonymized version of the data is available at https://osf.io/sny8j/.
